# Insulin Resistance at the Crossroads of Metabolic Inflammation, Cardiovascular Disease, Organ Failure and Cancer

**DOI:** 10.3390/biom15121745

**Published:** 2025-12-17

**Authors:** Amedeo Lonardo, Ralf Weiskirchen

**Affiliations:** 1 Ospedale Civile di Baggiovara (-2023), Department of Internal Medicine, AOU Modena, 41100 Modena, Italy; 2 Institute of Molecular Pathobiochemistry, Experimental Gene Therapy and Clinical Chemistry (IFMPEGKC), RWTH University Hospital Aachen, Pauwelsstr. 30, D-52074 Aachen, Germany; rweiskirchen@ukaachen.de

**Keywords:** cancer, cirrhosis, CKD, dementia, heart failure, MACE, MASLD, metaflammation, non-communicable disease, obesity, pathobiology, type 2 diabetes

## Abstract

Insulin resistance (IR) describes impaired hormone signaling that triggers compensatory homeostatic responses resulting in hyperinsulinemia, increased accumulation of fatty substrates, lipotoxicity, oxidative stress, inflammation, cell death and fibrosis in target tissues. These processes ultimately lead to organ dysfunction and predispose certain individuals to various types of cancer. In this context, we will review the molecular pathogenesis and clinical significance of IR, its role in ‘metaflammation’, and the damage caused by IR in the pancreas, cardiovascular system, liver, and kidneys. Additionally, we will discuss principles of drug treatment for IR and outline a research agenda in this field.

## 1. Introduction

Insulin resistance (IR) can be defined as a state where abnormally high levels of insulin are needed to produce a normal response in target tissues [1,2]. According to Aronis and Mantzoros [3], who have extensively studied this topic, IR was first identified by Rosalyn S. Yalow and Solomon A. Berson in 1960. These authors, using a new method to measure insulin, found that individuals with late-onset diabetes mellitus had elevated insulin levels [1,2]. The next breakthrough came when it was discovered that in rodents, high insulin levels due to IR were associated with abnormal insulin binding to its receptor [4,5]. In 1976, the first evidence that defects in the insulin receptor could lead to IR in humans provided further confirmation of the significance of IR in human health [6]. In 1988, Reaven proposed that IR was the underlying factor in the modern concept of metabolic syndrome, which includes high blood pressure, abnormal blood sugar levels, and unhealthy cholesterol levels [7].

**Figure 1:**
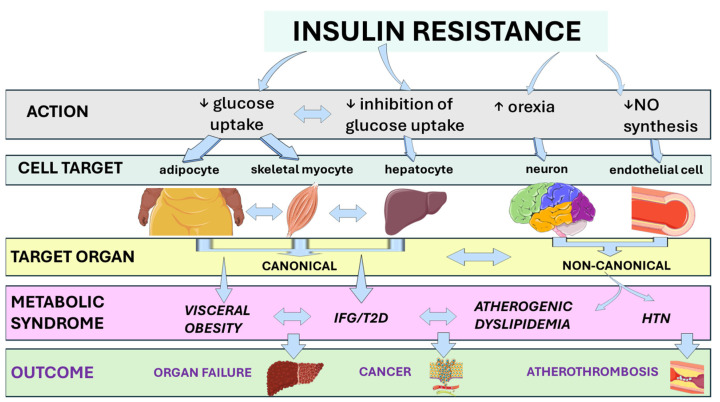
From insulin resistance to adverse clinical outcomes via the metabolic syndrome. Insulin resistance drives metabolic dysfunction by acting on cell targets and target organs. This systemic cascade of events will eventually contribute to an increased risk of T2D and other features of the metabolic syndrome, as well as impaired cardio-metabolic-hepato-renal health and potentially severe clinical outcomes in a subset of individuals. Abbreviations: HTN, arterial hypertension; IFG, impaired fasting glucose; NO, nitric oxide; T2D, type 2 diabetes. This original illustration was created using Servier Medical ART (SMART) and is licensed under the Creative Commons Attribution 4.0 International License (CC BY 4.0).

Figure 1 illustrates the link between IR and clinical outcomes through the metabolic syndrome [8,9,10,11,12,13].

Over the past few decades, research has expanded the understanding of IR beyond type 2 diabetes (T2D) and metabolic syndrome. It now highlights that IR plays a crucial role as a strategic intersection among various seemingly unrelated conditions. Complex pathophysiological systemic mechanisms, such as cell stress, mitochondrial dysfunction, and chronic subclinical low-grade inflammation, often exacerbated by factors like obesity, are intricately linked with IR. This, in turn, increases the risk of cardiovascular disease (CVD), organ failure, and various cancer types by triggering a vicious cycle where each metabolic dysfunction worsens and perpetuates other features of the metabolic syndrome over time [14,15,16,17].

IR, a modern epidemic reflecting a complex interplay between lifestyle habits and psycho-social determinants, imposes a significant financial burden. This is mainly due to the increased rates of T2D, CVDs, and metabolic dysfunction-associated steatotic liver disease (MASLD) [18,19,20]. The ongoing need for treatment drives up healthcare costs and results in productivity losses, impacting individuals, businesses, and the economy. Moreover, IR associated with obesity has serious social implications, including stigma and discrimination, which can significantly affect mental health and reinforce socio-economic disparities [19].

With this background, the present review synthesizes current knowledge on the molecular pathogenesis of IR, elucidates its role in metaflammation and the progression to CVD, organ failure, and cancer. We also propose a translational agenda aimed at improving prevention and treatment strategies.

## 2. Research Strategy

Following the objectives outlined in the Introduction, we conducted a comprehensive narrative literature search in PubMed, Embase, Web of Science, and Scopus for articles published up to October 2025. We used key terms such as “insulin resistance,” “metaflammation,” “metabolic syndrome,” “type 2 diabetes,” “cardiovascular disease,” “organ failure,” and “cancer” to guide our search. Additionally, we searched reference lists of important articles to identify any additional relevant studies. Our priority was to include original research, large cohort studies, randomized controlled trials, meta-analyses, and authoritative reviews written in English. We also considered studies focusing on pediatric populations, gestational diabetes, or rare monogenic disorders, if they provided insights that could be applied to adult metabolic diseases. We carefully screened titles, abstracts, and full texts of selected studies. Information regarding study design, population characteristics, molecular findings, clinical outcomes, and therapeutic implications was extracted. This information was then qualitatively synthesized to provide an integrative overview that informs the subsequent sections of this review.

## 3. Etiology and Assessment of Insulin Resistance

### 3.1. Causes of Insulin Resistance

There are numerous causes that can eventually lead to the development of IR. Genetic forms of IR include myotonic dystrophy, ataxia-telangiectasia, Alstrom syndrome, Rabson-Mendenhall syndrome, Werner syndrome, lipodystrophy, type-A IR caused by abnormalities of the insulin receptor gene, which can result in abnormal glucose homeostasis, ovarian virilization, and acanthosis nigricans, and type-B IR triggered by insulin receptor autoantibodies leading to altered glucose homeostasis, ovarian-type hyperandrogenism, and acanthosis nigricans [21]. Table 1 summarizes the acquired forms of IR.

### 3.2. Assessment of Insulin Resistance in Clinics and in Epidemiological Studies

In clinical practice, IR should be suspected in individuals with a family history of T2D, especially if objective findings indicate an enlarged waist circumference, arterial hypertension, or acanthosis nigricans [56]. Another clinical clue to IR is the presence of polycystic ovary syndrome (PCOS) [57]. PCOS may be suspected in young women based on altered menses, hyperandrogenism and ultrasonographic evidence of polycystic ovaries [58].

The simplest laboratory test to reveal IR is assessing fasting plasma glucose [59], and elevated fasting glycemia may be an early and inexpensive biomarker of IR, particularly when associated with concomitant compensatory fasting hyperinsulinemia [60]. Although the significance of post-prandial hyperglycemia is widely acknowledged in the context of diabetes, its impact in non-diabetic subjects is poorly defined [61]. The oral glucose tolerance test (OGTT), while used primarily to diagnose glucose intolerance, by measuring blood glucose and insulin levels at intervals after a glucose oral load, can offer insights into glucose tolerance and insulin dynamics [62].

The triglyceride-glucose (TyG) index is calculated using fasting triglycerides and fasting blood glucose according to the formula *ln(triglycerides x glucose*/2/*triglycerides*×*glucose*/2) and is a simple, cost-effective, and reliable surrogate bio marker in the diagnosis of IR [63]. Although it is correlated with standard measures such as the Homeostatic Model Assessment of Insulin Resistance (HOMA-IR) [64] and is also used to assess cardiometabolic risk [65]. Its accuracy and optimal cutoff values vary depending on age, race, and sex. A study based on more than 2000 hypertriglyceridemic adults has suggested a single cutoff point of 4.5 to classify individuals with IR [66]. The HOMA-IR is a widely used simple surrogate index of IR that does not involve a log transformation and is simply calculated as the product of fasting insulin and glucose [67]. A Korean study enrolling 10,997 participants found that the cut-off values of the HOMA-IR were 2.20 in men, 2.55 in premenopausal women, and 2.03 in postmenopausal women [68].

The Quantitative Insulin Sensitivity Check Index (QUICKI) is another simple index that uses fasting glucose and insulin levels, differing from HOMA-IR. QUICKI uses a mathematical transformation, specifically the inverse of the sum of the logarithms of fasting insulin and glucose [69].

### 3.3. Assessment of Insulin Resistance in the Diabetes Research Setting

The hyperinsulinemic-euglycemic clamp, considered the gold standard in research, involves infusing insulin to suppress glucose production in the liver while simultaneously infusing a glucose solution to keep blood glucose levels stable and normal [70]. The rate of glucose infusion needed to maintain this stability during the clamp provides a precise measurement of insulin sensitivity [70].

Compared to the hyperinsulinemic-euglycemic clamp, the insulin tolerance test (IST) is a simpler yet highly accurate research-based test to determine IR [71]. The IST involves the intravenous infusion of glucose while monitoring its disappearance over time until it returns to fasting levels in order to estimate insulin sensitivity [59].

## 4. Molecular Physiology of Insulin Signaling and Mechanisms of Insulin Resistance

### 4.1. Inter-Organ Crosstalk: Hepatokines, Myokines, and Adipokines

Insulin action begins with receptor autophosphorylation, IRS engagement, PI3K activation, and downstream Akt-mediated effects that promote glucose uptake, glycogen synthesis, lipogenesis, and protein translation [72]. While this proximal cascade is ubiquitous, its systemic outcome is sculpted by a dynamic three-way dialogue among the liver, skeletal muscle, and adipose tissue. In the post-prandial state, insulin suppresses hepatic gluconeogenesis, stimulates glycogen storage, and drives de novo lipogenesis. However, the liver also acts as an endocrine organ, releasing hepatokines such as fetuin-A, fibroblast growth factor-21, and sex hormone–binding globulin (SHBG) that modify insulin responsiveness in distant tissues [73]. For example, Fetuin-A binds to the insulin receptor and toll-like receptor 4 (TLR4) on adipocytes and myocytes, promoting serine phosphorylation of IRS proteins and thereby dampening PI3K-Akt signaling. On the other hand, FGF-21 exerts the opposite effect by enhancing fatty-acid oxidation and glucose uptake, illustrating how shifts in hepatokine balance can tip systemic insulin sensitivity in either direction.

Skeletal muscle, which ordinarily disposes of approximately 70% of meal-derived glucose, secretes myokines whose profiles depend on contractile activity. Exercise releases irisin and myonectin that augment hepatic β-oxidation and promote browning of white fat [74,75]. Conversely, physical inactivity elevates myostatin, which suppresses GLUT4 expression and encourages adiposity, reinforcing IR [76]. Adipose tissue adds a third endocrine axis, in which anti-inflammatory adiponectin activates AMPK and PPAR-α in the liver and muscle to increase fatty acid combustion. However, its concentrations fall in obesity, while leptin, resistin, and visfatin rise, driving NF-κB and JNK activation in macrophages and parenchymal cells, thereby amplifying systemic IR [77].

### 4.2. Lipotoxicity and the Self-Expanding Network of the Metabolic Syndrome

When caloric intake chronically exceeds oxidative needs, subcutaneous fat deposits reach their storage limit. Surplus fatty acids are then redirected to visceral fat, the liver, muscles, pancreas, heart, and kidneys, where they accumulate as diacylglycerols, ceramides, and acyl-CoAs. These substances poison insulin signaling, a process termed lipotoxicity. These lipid intermediates activate novel protein kinase C (PKC) isoforms and TLR4, leading to IKKβ- and JNK-mediated inhibitory phosphorylation of insulin receptor substrate 1/2 (IRS1/2) and uncoupling of endothelial nitric-oxide synthase [78,79]. This, in turn, blunts insulin-mediated glucose transport, causes endothelial dysfunction, and raises blood pressure.

**Figure 2:**
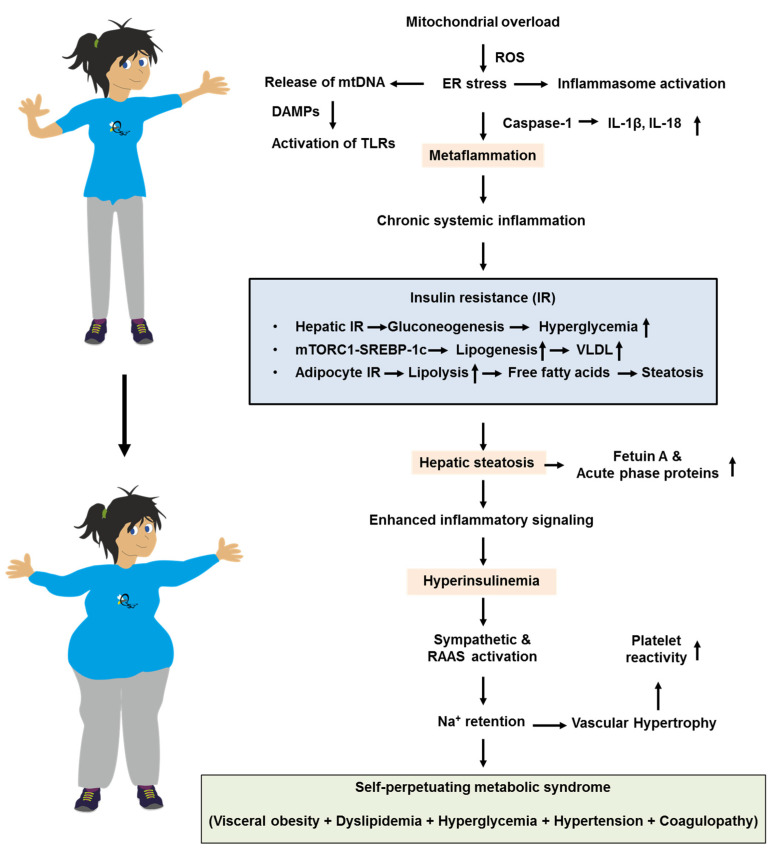
Simplified pathophysiological feedback loops that drive the progression of metabolic syndrome from metaflammation to hepatic steatosis and hyperinsulinemia. For more information, please refer to the text. Abbreviations: DAMPs, damage-associated molecular patterns; ER, endoplasmic reticulum; IL-1β, interleukin-1β; IL-18, interleukin 18; mtDNA, mitochondrial DNA; RAAS, renin–angiotensin–aldosterone system; ROS, reactive oxygen species; TLR(s), toll-like receptors; VLDL, very low-density lipoproteins.

Mitochondrial overload generates release of mitochondrial DNA (mtDNA) that acts as damage-associated molecular patterns (DAMPs), leading to activation of toll-like receptors (TLRs). It further leads to reactive oxygen species (ROS) formation that intensifies ER stress and inflammasome activation. This leads to caspase-1–dependent maturation of IL-1β and IL-18, resulting in “metaflammation” that perpetuates cytokine spill-over. This creates a positive feedback loop that maintains tissues in a state of chronic IR. Selective hepatic IR further skews metabolism [80]. While Akt inhibition allows unabated gluconeogenesis and fasting hyperglycemia, mTORC1-driven SREBP-1c activity remains relatively preserved [81]. This sustains de novo lipogenesis and VLDL overproduction, raising plasma triglycerides, lowering HDL cholesterol via cholesteryl ester transfer protein (CETP) exchange, and accelerating atherogenesis [82]. Simultaneously, impaired insulin suppression of adipocyte lipolysis floods the portal circulation with free fatty acids, worsening hepatic steatosis. This fuels the production of fetuin A, ceruloplasmin, and acute-phase reactants that spread inflammatory signals. These events might trigger a cascade in which the metaflammation provokes IR and hepatic steatosis, finally ending in metabolic syndrome (Figure 2).

Hyperinsulinemia, originally a compensatory response, exacerbates sympathetic activation and renin–angiotensin–aldosterone signaling [83]. This drives sodium retention and vascular hypertrophy, while directly stimulating smooth-muscle proliferation and platelet reactivity. These factors integrate hypertension and pro-thrombotic risk into the metabolic-syndrome complex [83]. Common molecular lesions, such as ectopic lipid overload, organelle stress, and inflammatory kinase activation, act as the centripetal force that clusters visceral obesity, dyslipidemia, hyperglycemia, hypertension, and pro-coagulant states. This causes the syndrome to perpetuate and worsen progressively over time.

### 4.3. Drug-Induced Derailment of Insulin Signaling

A variety of therapeutic agents can exacerbate IR by targeting the same molecular nodes that regulate insulin action. Glucocorticoids increase the expression of enzymes involved in gluconeogenesis (PEPCK, G6Pase), inhibit the translocation of muscle GLUT4, and promote visceral fat accumulation, leading to hyperinsulinemia and hyperglycemia [83]. Calcineurin inhibitors like cyclosporine and tacrolimus hinder the dephosphorylation of nuclear factor of an activated T cell (NFAT), reducing insulin production and β-cell mass, while also decreasing muscle glucose uptake seen in post-transplant diabetes [84,85].

HIV protease inhibitors interfere with GLUT4 function and cause lipodystrophy, ectopic fat storage, and oxidative stress, resulting in severe IR similar to metabolic syndrome [86,87]. Atypical antipsychotics affect central appetite regulation through 5-HT2C and H1 blockade but also impair mitochondrial function in the liver and β-cells, disrupt AMPK signaling, increase fat production, leading to weight gain, dyslipidemia, and poor blood sugar control [88]. Selective serotonin-reuptake inhibitors, while typically weight-neutral, can increase adrenergic activity and cortisol release, reducing insulin effectiveness. Androgen-deprivation therapy reduces muscle mass and increases visceral fat, raising the risk of diabetes in men with prostate cancer. Even high doses of exogenous insulin can down-regulate its own receptor and promote fat formation, worsening IR and requiring higher doses, known as “hyperinsulinemia-induced IR”.

Understanding these mechanisms is crucial to make informed decisions on drug selection and potentially combine with insulin-sensitizing medications like metformin, thiazolidinediones, GLP-1 receptor agonists, or sodium-glucose transporter 2 (SGLT2) inhibitors to mitigate iatrogenic metabolic harm while maintaining treatment effectiveness. IR arises from disrupted signaling in liver, muscle, and fat cells, exacerbated by lipotoxic and inflammatory factors that link these tissues, and exacerbated by medications targeting overlapping pathways. This understanding supports a comprehensive, tissue-specific, and drug-conscious approach to combat the complex metabolic syndrome.

## 5. Pathobiology and Clinical Science Associating Ethnicity, Insulin Resistance, Sex and Aging

### 5.1. Pathobiology Associating Insulin Resistance, Ethnicity Sex and Aging

Marked heterogeneity in the prevalence and severity of IR across ethnic groups, between the sexes and over the life span reflects a mosaic of molecular differences that modulate the canonical insulin signaling cascade. Genome-wide association studies indicate that risk alleles in loci such as *TCF7L2*, *PPARG*, *IRS1* and *SLC16A11* are differentially distributed among populations of African, Hispanic, South-Asian and East-Asian ancestry, predisposing carriers to impaired insulin receptor substrate (IRS) phosphorylation and reduced glucose uptake at lower body-mass indices than in populations of European descent [89]. Moreover, Asians and people of African ancestry tend to accumulate more visceral and hepatic fat and possess smaller adipocytes that reach their lipid storage threshold earlier, promoting ectopic diacylglycerol and ceramide deposition, activating novel PKC isoforms and phosphorylation of IRS1/2, thereby amplifying IR at comparatively modest degrees of obesity [90]. Ethnic variation in adipokine profiles, such as lower adiponectin and higher resistin, visfatin and fetuin-A levels, further augments inflammatory IKKβ/JNK signaling and fuels metaflammation, providing a mechanistic explanation for the high IR burden seen in Black and Hispanic communities.

Sexual dimorphism in IR is driven chiefly by sex hormones and their influence on adipose topography, mitochondrial biology and immune tone [91]. Estrogen activates ERα, boosts insulin-stimulated Akt phosphorylation and promotes subcutaneous rather than visceral fat deposition; it also enhances mitochondrial biogenesis via PGC-1α and dampens NF-κB activity, conferring pre-menopausal women relative protection against IR [92]. Men, with higher androgen but lower estrogen exposure, exhibit larger myofiber cross-sectional areas and preferential visceral fat expansion, conditions that favor free fatty-acid flux to the liver and skeletal muscle, propagating lipotoxic inhibition of insulin signaling [92]. After menopause, declining estrogen, rising FSH and augmented visceral adiposity converge to reduce adiponectin, elevate leptin and accentuate pro-inflammatory cytokine release, narrowing the sex gap in IR prevalence [92]. Conversely, hyperandrogenism in women with PCOS and hypogonadism in aging men impair GLUT4 translocation and mitochondrial oxidative capacity, reinforcing IR in a hormone-specific manner.

Aging compounds these disparities through progressive sarcopenia, mitochondrial DNA damage, altered mitophagy and redistribution of fat from subcutaneous to ectopic depots [93]. This enhances ROS generation and stimulates *NLRP3* inflammasome activity, thereby intensifying IRS serine phosphorylation and diminishing PI3K–Akt efficiency. Age-related clonal hematopoiesis and immunosenescence bias the cytokine milieu toward chronic low-grade inflammation, while epigenetic drift and telomere attrition exacerbate metabolic inflexibility, creating a milieu in which even modest genetic or hormonal vulnerabilities translate into overt IR. Thus, the intersection of ethnic/genetic background, sex hormones and age-linked cellular stress orchestrates a spectrum of molecular perturbations, including lipotoxicity, inflammatory kinase activation, and organelle dysfunction, which underlie the observed demographic gradients in IR.

### 5.2. Clinical Science Associating Insulin Resistance, Ethnicity, Sex, and Aging

IR is associated with ethnicity, sex and aging, with each factor playing a complex and interacting role. Certain ethnic groups such as Black, Hispanic individuals, and Asians, have higher rates of IR compared to non-Hispanic Whites, even at the same body weight or body mass index (BMI) [94]. This can be linked to factors such as genetics, body fat distribution, and carbohydrate metabolism [95].

Aging-associated worsening of the odds of IR is deemed to be due to age-related increases in adiposity [96]. Additional factors explaining the increased risk of IR with aging include a decline in muscle mitochondrial function, and the significant increase in the metabolic syndrome in both sexes after the age of 60 [97].

Sex differences are also significant, with pre-menopausal women generally being more insulin sensitive than men. This protection diminishes after menopause due to the decline of estrogen levels and expanded visceral adiposity [98,99]. Sex hormones influence insulin sensitivity differently in men and women. For example, hyperandrogenemia in women, typically observed, among individuals with polycystic ovary syndrome (PCOS), and hypogonadism in men (which may result from aging, diabetes or cirrhosis) are both linked with IR and MASLD risk [99].

Interestingly, indices of IR exhibit a sex-specific predictive ability, with TyG-BMI calculated as the natural logarithm (fasting triglyceride [mg/dL] × fasting glucose) [100] being the only index able to predict functional decline in women and HOMA-IR in men [101]. These findings suggest that a cause-and-effect mutual relationship links aging and IR in a sex-specific manner.

## 6. Pathobiology and Clinical Science Associating Insulin Resistance and Metabolic Inflammation

### 6.1. Pathobiology Associating Insulin Resistance and Metabolic Inflammation

Visceral adipocyte hypertrophy, hypoxia and mechanical stretch create an initial dangerous environment that recruits monocytes and converts tissue-resident M2 macrophages into pro-inflammatory M1 cells [102], establishing the earliest focus of “metaflammation” in insulin-resistant states. Saturated fatty acids released from these stressed adipocytes ligate TLR4 on adipocytes, hepatocytes and macrophages, triggering MyD88–IRAK–IKKβ and JNK cascades that serine-phosphorylate IRS proteins, directly antagonizing PI3K-Akt signaling and propagating IR. Simultaneously, mitochondrial ROS, ceramides and extracellular ATP activate the NLRP3 inflammasome, driving caspase-1-dependent maturation of IL-1β and IL-18, cytokines that amplify both local and systemic insulin antagonism [103]. Lipid spill-over into the liver and skeletal muscle yields diacylglycerols and ceramides that activate novel PKC isoforms and provoke endoplasmic-reticulum stress, each feeding back into JNK/NF-κB/AP-1 transcriptional programs that sustain cytokine output while inhibiting insulin-stimulated glucose transport and glycogen synthesis. Compensatory hyperinsulinemia further fuels the loop by up-regulating SREBP-1c and ChREBP, expanding lipid stores and generating a constant supply of lipotoxic intermediates that keep innate immune sensors engaged [104]. Adipokine imbalance adds another layer: excess leptin, resistin and visfatin potentiate Th1 polarization and macrophage activation, whereas hypoadiponectinemia removes AMPK- and PPAR-α-mediated anti-inflammatory brakes. Hepatokines such as fetuin-A synergize with saturated fatty acids to activate TLR4 [105], and myokines like myostatin curtail insulin-sensitizing irisin release [106], broadcasting inflammatory cues across metabolic organs. The result is a self-perpetuating, low-grade inflammatory circuit (i.e., metaflammation) that not only entrenches IR but also primes endothelial cells for dysfunction, β-cells for failure and parenchymal organs for fibrosis, thereby constituting the molecular nexus linking nutrient surplus to cardio-metabolic-hepato-renal disease.

### 6.2. Clinical Science Associating Insulin Resistance and Metabolic Inflammation

Visceral obesity promotes chronic, low-grade subclinical sterile inflammation, often referred to as *“metaflammation”* [107]. Strong epidemiological and clinical evidence supports the idea that expanded and inflamed adipose tissue in individuals with obesity shows a shift in immune cells from anti-inflammatory M2 macrophages to pro-inflammatory M1 macrophages, a pattern linked to IR [108]. Obesity is also characterized by elevated levels of IR-inducing adipokines such as leptin, visfatin, and resistin [107,108]. With global obesity rates on the rise, projections indicate that by 2030, severe obesity will be most prevalent among low-income adults, Black individuals, and women, highlighting the urgent need for effective intervention strategies [109]. In turn, IR is marked by secondary compensatory hyperinsulinemia, which can perpetuate inflammation and promote tissue damage through the accumulation of ectopic fat [110].

The likelihood of subclinical systemic inflammation due to IR can vary, but studies consistently show a significant and positive association. For example, one study found that a higher HOMA-IR score was linked to a significantly higher likelihood of subclinical inflammation, as measured by high-sensitivity C-reactive protein (hs-CRP) among prediabetic individuals with MASLD [111]. Another study demonstrated that elevated IR (higher HOMA-IR) increased the risk of future IR and T2D, with a combined higher risk of inflammation and IR [112]. Overall, these studies support the idea that IR is strongly connected to systemic subclinical inflammation, suggesting that treating IR could effectively reduce the systemic inflammatory state in individuals with metabolic dysfunction.

## 7. Pathobiology and Clinical Science Associating Insulin Resistance and Cardiovascular Disease

### 7.1. Pathobiology Associating Insulin Resistance and Cardiovascular Disease

IR triggers a selective impairment of the endothelial PI3K-Akt pathway, leading to decreased phosphorylation of endothelial-nitric-oxide synthase (eNOS) and a significant reduction in bioavailable nitric oxide (NO), a key vasodilator and antithrombotic molecule [113]. The parallel MAPK arm of insulin signaling remains relatively unaffected, promoting the release of endothelin-1 and the proliferation of vascular smooth muscle cells. This shifts the vasculature towards vasoconstriction, sodium retention, and sympathetic activation, all crucial factors in the development of arterial hypertension in states of IR. The uncoupling of eNOS, caused by mitochondrial ROS and tetrahydrobiopterin depletion, transforms the enzyme from an NO generator to a superoxide producer [114]. This amplifies oxidative stress, further reducing NO availability, leading to the stiffening of resistance arteries and an increase in systemic blood pressure.

The decreased NO levels also create a pro-atherothrombotic environment, in which reduced anti-adhesive signaling boosts the expression of endothelial VCAM-1 and ICAM-1, facilitating the migration of leukocytes [115]. Meanwhile, impaired Akt–eNOS activity allows the pro-atherogenic NF-κB cascade to go unchecked, resulting in the secretion of inflammatory molecules like IL-6, MCP-1, and tissue factor. Hyperinsulinemia increases hepatic VLDL output and generates small, dense LDL particles that are easily oxidized. These particles, along with elevated free fatty acids and ceramides, activate TLR4 and NLRP3 inflammasomes in macrophages, leading to foam-cell formation and unstable plaques [116]. Platelet hyper-reactivity, caused by low NO and prostacyclin, high PAI-1, and increased thromboxane A_2_ synthesis, contributes to a pro-thrombotic state that accelerates adverse cardiovascular events in individuals with IR.

In the myocardium, chronic IR reduces insulin-stimulated glucose uptake, forcing cardiomyocytes to rely on fatty acid oxidation, a process that increases mitochondrial ROS and accumulates lipotoxic intermediates, leading to glucolipotoxicity, cell apoptosis and fibrosis [117]. Endothelial NO deficiency impairs endothelial dysfunction, coronary microvascular dilation, causing sub-endocardial hypoxia that affects diastolic relaxation and promotes the development of heart failure with preserved ejection fraction (HFpEF) [118,119]. Additionally, hyperinsulinemia activates the sympathetic nervous system and renin–angiotensin–aldosterone axis, resulting in concentric left-ventricular hypertrophy and extracellular-matrix deposition, changes that can progress to overt systolic dysfunction if the metabolic insult persists [120].

Thus, through interconnected mechanisms involving eNOS dysfunction, oxidative stress, and lipotoxic-inflammatory signaling, IR intricately links arterial hypertension, atherothrombosis, and heart-failure phenotypes along a shared molecular continuum.

**Figure 3:**
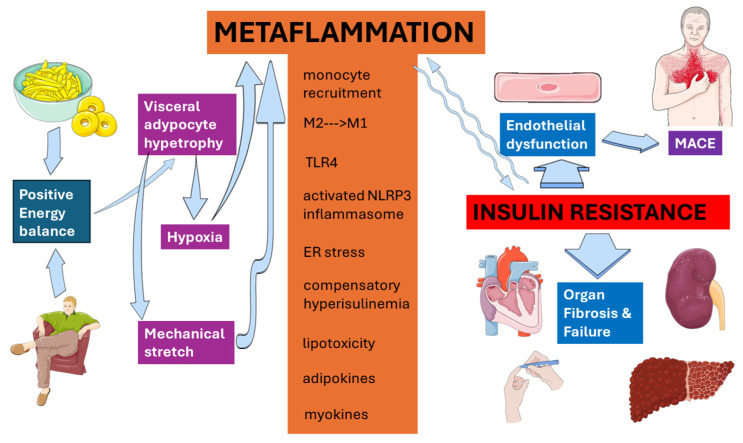
Metaflammation at a glance. Metaflammation serves as the crucial link connecting enlarged dysfunctional visceral adipose tissue with negative clinical consequences. This original illustration, based on references cited in the text, was created using Servier Medical ART (SMART) and is licensed under the Creative Commons Attribution 4.0 International License (CC BY 4.0).

Figure 3 schematically summarizes the principal steps connecting positive energy balance with cardiovascular, metabolic, and hepatorenal outcomes through metaflammation.

### 7.2. Clinical Science Associating Insulin Resistance and Cardiovascular Disease

IR is a major risk factor for cardiometabolic disorders, including hypertension and atherosclerosis. Individuals living with the prototypic state of IR, namely T2D, are typically exposed to an increased risk for macrovascular complications, which represent the major cause of mortality in this population [121]. However, effective treatment of established cardiovascular risk factors such as dyslipidemia, arterial hypertension, and a procoagulant state will still leave a significant amount of residual, unexplained cardiovascular risk. This risk is contributed to by persistent IR via endothelial dysfunction, dyslipidemia, and a systemic pro-inflammatory state that promotes the buildup of plaque in arteries, increasing the risk of major adverse cardiovascular events (MACE) [122].

Two seminal studies have addressed the importance of lifestyle changes in T2D: the Look AHEAD trial-a large, randomized clinical study comparing an intensive lifestyle intervention with a standard diabetes support and education control group for overweight and obese adults with T2D [123]-and the Italian Diabetes and Exercise Study, which showed that supervised exercise comprising aerobic and resistance training is more effective than standard exercise counseling alone for improving cardiovascular risk factors in individuals with T2D and the metabolic syndrome [124]. Together, these trials demonstrate that lifestyle management notably enhances physical fitness, glycemic homeostasis assessed with HbA1c, and coronary heart disease risk factors [121].

Glucose-lowering therapies significantly contribute to reducing cardiovascular risk in individuals with T2D [125]. Additionally, bariatric surgery can enhance cardiovascular outcomes by promoting weight loss and reducing glycated hemoglobin levels [126]. Lastly, a comprehensive approach to managing CVD risk factors, including addressing blood glucose levels, smoking, dyslipidemia, and high blood pressure, helps lower mortality rates from MACE [127]. Collectively, these various studies consistently show that IR is causally involved in the development and progression of CVD.

## 8. Pathobiology and Clinical Science Associating Insulin Resistance and Organ Failure

### 8.1. Pathobiology Associating Insulin Resistance and Organ Failure

Persistent IR forces pancreatic β-cells to sustain supraphysiological insulin output. Initially, this is achieved through β-cell hyperplasia, increased insulin-gene transcription, and augmented secretory granule biogenesis, mechanisms that compensate for diminished peripheral insulin action. Chronic energy surplus, however, exposes β-cells to glucolipotoxicity [128]. Elevated glucose boosts mitochondrial ROS, while free fatty acid influx generates toxic diacylglycerols and ceramides that activate PKCε, JNK, and endoplasmic reticulum (ER) stress pathways [129,130]. This culminates in oxidative damage, unfolded-protein response exhaustion, and ultimately apoptosis or dedifferentiation of β-cells to a non-secretory state. Lineage-tracing studies in rodents and analyses of human autopsy islets corroborate a trajectory in which initial β-cell expansion gives way to progressive mass and function loss once fasting glycaemia rises above ~5.5 mmol/L, defining the transition from compensated IR to overt T2D.

Beyond the pancreas, ectopic deposition of lipids in non-adipose tissues provides the biochemical substrate for organ dysfunction. In the liver, incomplete β-oxidation of oversupplied fatty acids yields ROS and lipid peroxides that provoke hepatocyte death and activate Kupffer cells [131]. The ensuing secretion of TNF-α, IL-6, and TGF-β stimulates stellate cell transdifferentiation and collagen deposition, driving the evolution from simple steatosis to MASH and cirrhosis. In the kidney, podocyte and proximal-tubule lipid overload induces ER stress and NLRP3-inflammasome activation, promoting mesangial expansion, glomerulosclerosis, and progressive CKD that ultimately manifests in nearly 40% of individuals with long-standing T2D. Cardiomyocytes exposed to high circulating fatty acids switch from glucose to fatty-acid oxidation, an energetically costly process that accelerates mitochondrial ROS generation and accumulates lipotoxic intermediates and DNA damage [132]. This triggers apoptosis, interstitial fibrosis via TGF-β/SMAD signaling, and ventricular stiffening characteristics of heart failure with preserved ejection fraction. In the brain, insulin-signaling defects impair neuronal glucose uptake, facilitate tau hyperphosphorylation, and reduce Aβ clearance. Microglial inflammasome activation perpetuates neuroinflammation, linking IR to cognitive decline and Alzheimer’s disease phenotypes.

These degenerative cascades are fueled by systemic immunometabolic derangements inherent in IR. Visceral adipose expansion shifts macrophage polarity from M2 to M1, elevating TNF-α, IL-6, and MCP-1. These cytokines enter the circulation and impair insulin-receptor signaling in distant tissues through IKKβ- and JNK-mediated serine phosphorylation of IRS1/2. Hyperinsulinemia itself exerts pro-inflammatory effects by activating NF-κB and upregulating VCAM-1 expression, whereby adhesion of leukocytes and monocytes to the endothelium increases [132]. Excess leptin and reduced adiponectin further skew the immune milieu toward Th1 and Th17 dominance, lowering the threshold for tissue injury. In parallel, hepatic release of fetuin-A complexes with saturated fatty acids to engage TLR4 on Kupffer cells and renal macrophages, amplifying inflammasome activation and fibrosis. Endothelial NO deficiency, secondary to selective insulin-signaling defects, enhances oxidative stress, recruits neutrophils, and facilitates platelet activation, a triad that not only accelerates atherothrombosis but also compromises organ perfusion, thereby worsening cardiac, renal, and cerebral outcomes [118,119].

Collectively, IR initiates a multidimensional assault. It begins with β-cell exhaustion, which erodes insulin supply. This leads to the production of lipotoxic intermediates that trigger parenchymal apoptosis and fibrogenesis, as well as chronic low-grade inflammation [133]. This inflammation orchestrates immune cell infiltration and excess cytokines, establishing a self-reinforcing loop. Ultimately, this culminates in the failure of the pancreas, liver, kidney, heart, and brain.

### 8.2. Clinical Science Associating Insulin Resistance and Organ Failure

Over time, chronic inflammation and metabolic derangements closely linked to IR can result in organ damage and failure. For example, IR and chronic subclinical inflammation play crucial roles in the development of pancreatic β-cell failure and T2D [134]. While T2D is mainly caused by decreased insulin secretion from β-cells in individuals with long-standing IR, the connection between reduced β-cell mass and dysfunction remains a topic of debate. Studies in mice using lineage tracing suggest that β-cell mass can increase to compensate for IR, sustaining insulin secretion until hyperglycemia occurs through β-cell hyperplasia and activation of various cellular mechanisms, such as islet cell trans-differentiation and dedifferentiation to a non-secretory state [134]. This adaptive physiological hyperinsulinemia typically prevents the onset of T2D in most individuals. However, some may eventually develop overt hyperglycemia due to β-cell failure to compensate [135]. Nonetheless, the majority of human islet data is derived from autopsy or donor samples, which limits functional insights due to the absence of in vivo profiling data [136].

Longitudinal studies of individuals progressing to T2D demonstrate an elevation in insulin levels during both the normoglycemic and prediabetic stages. This elevation sustains near-normal glycemia despite IR, which is indicative of β-cell compensation, followed by a subsequent decline as fasting glycemia exceeds the upper normal limit of 5.5 mM (β-cell failure) [137]. Additionally, during the natural history of T2D progressive deterioration of β-cell function occurs associated with decreased β-cell mass due to cell apoptosis. This process is not invariably irreversible and may be blocked or delayed by effective disease management through lifestyle modification alone, eventually requiring insulin therapy at later stages of diabetic disease [138].

Similarly, in the pathogenesis of T2D, long-standing IR may eventually lead to liver cirrhosis via MASLD and metabolic dysfunction-associated steatohepatitis (MASH) in individuals living with either obesity or T2D [139,140]. A seminal population-based cohort study totaling 11,465 adults without any evidence of liver cirrhosis at the study entry or during the first 5 years of follow-up, who were followed-up for a mean of 12.9 years showed that among individuals who did not consume alcohol, there was a strong association between obesity (adjusted hazard ratio 4.1, 95% CI 1.4–11.4) or being overweight (adjusted hazard ratio 1.93, 95% CI 0.7–5.3) and cirrhosis-related death or hospitalization [141]. Consistent with these findings, an Australian study involving 8631 participants demonstrated that individuals with T2D compared to those without T2D had a significantly higher prevalence of cryptogenic cirrhosis (42.4% vs. 27.3%; *p* < 0.001), MASLD/MASH (13.8% vs. 3.4%; *p* < 0.001), and admissions for hepatocellular carcinoma (HCC) (18.0% vs. 12.2%; *p* < 0.001). Furthermore, among patients with liver cirrhosis, those with T2D experienced a significantly greater median hospital stay (6 [range, 1–11] vs. 5 [range, 1–11] days; *p* < 0.001), twice the rate of non-cirrhosis-related admissions (incidence rate ratio [IRR], 2.03; 95% confidence interval [CI], 1.98–2.07), a 1.35-fold increase in cirrhosis-related admissions (IRR, 1.35; 95% CI, 1.30–1.41), and markedly reduced survival rates (*p* < 0.001) [142]. This study clearly illustrates the notion that, in the context of MASLD, HCC is another complication of long-standing IR facilitated by the development of MASLD-cirrhosis. However, it may typically occur with the background of non-cirrhotic livers [143].

Similarly, the strain posed by obesity on renal function can lead to kidney failure. A meta-analysis of 21 articles totaling 3,504,303 individuals (521,216 with obesity) followed up for an average of 9.86 years found that the relative risk of obese people developing chronic kidney disease (CKD) was 1.81 (95%CI: 1.52–2.16), indicating that living with obesity carries a 1.81 times higher risk of developing CKD than the non-obese population [144]. Another meta-analysis of 20 studies totaling 1,711,926 participants found that around 40% of those living with T2D will develop CKD, with the risk increasing in parallel with the duration of the diabetic disease [145]. The presence of MASLD, common among individuals with diabesity, is considered an independent risk factor for developing CKD [146,147].

Additional examples of IR eventually leading to target organ damage include Alzheimer’s disease [148], MACE and heart failure [149,150]. Interestingly, fibrosing MASLD could be an intermediate step associated with IR and target organ damage in both cases [151,152]. In summary, strong evidence demonstrates that IR is a major determinant of organ failure across a range of clinical manifestations spanning dementia to cardiovascular and hepatorenal health, with MASLD playing a significant role in mediating the pathomechanics of target organ dysfunction.

## 9. Pathobiology and Clinical Science Associating Insulin Resistance and Cancer

### 9.1. Pathobiology Associating Insulin Resistance and Cancer

Hyperinsulinemia, secondary to IR, is a ‘silent killer’ that leads to chronically elevated levels of circulating insulin and C-peptide [153]. This drives the over-activation of the insulin receptor-A (IR-A) and hybrid IR/IGF-1 receptors, which strongly couple to the mitogenic MAPK and mTOR–S6K axes [154,155]. This stimulation results in uncontrolled cellular proliferation and inhibits apoptosis, creating an environment in which neoplastic clones can easily expand. At the same time, high insulin suppresses the hepatic synthesis of SHBG, leading to increased levels of free estradiol and testosterone [156]. These unbound hormones act on estrogen or androgen receptors in breast, ovarian, endometrial, and prostate tissues, promoting cell cycle progression and genomic instability. This explains the sexual dimorphism observed in obesity and diabetes-related cancers. In a study conducted by Schrijnders et al., comprising 69,583 patients with T2D from a primary care cohort database, it was found that T2D is seemingly associated with a significantly higher risk of obesity-related cancer in women compared to men [157]. Furthermore, this risk is already elevated years before the diagnosis of diabetes in women. This study supports the idea that T2D diminishes the relative advantage that women typically have in the general population regarding cancer risk.

IR-driven lipotoxicity further fuels oncogenesis by causing the ectopic accumulation of saturated fatty acids, diacylglycerols, and ceramides. This activates PKC, NF-κB, and JNK, enhancing the transcription of pro-inflammatory cytokines like TNF-α and IL-6. These cytokines create a tumor-permissive micro-environment rich in ROS and growth factors, inducing DNA damage, epigenetic reprogramming, and epithelial-to-mesenchymal transition (EMT) [158]. This fosters both the initiation and invasion of malignant cells. Chronic activation of the NLRP3 inflammasome and secretion of IL-1β and IL-18 stimulate angiogenesis via VEGF up-regulation, providing tumors with the vascular support needed for expansion and metastasis.

Dysregulation of adipokines adds another oncogenic layer. Elevated leptin enhances JAK-STAT3 and PI3K-Akt signaling in neoplastic cells, promoting proliferation and inhibiting apoptosis. On the other hand, hypoadiponectinemia removes AMPK-mediated restraints on mTOR and ERK pathways, tipping the balance toward tumor growth. Hepatic steatosis, a hallmark of MASLD, increases the secretion of pro-oncogenic hepatokines such as fetuin-A and angiopoietin-like protein 8 (ANGPTL8), often in complex with ANGPTL3 or ANGPTL4 [159,160]. Together with insulin-induced FGF-2 and PDGF release, these potentiate systemic insulin-like and angiogenic signals that accelerate hepatocarcinogenesis and may nurture distant metastases.

**Figure 4:**
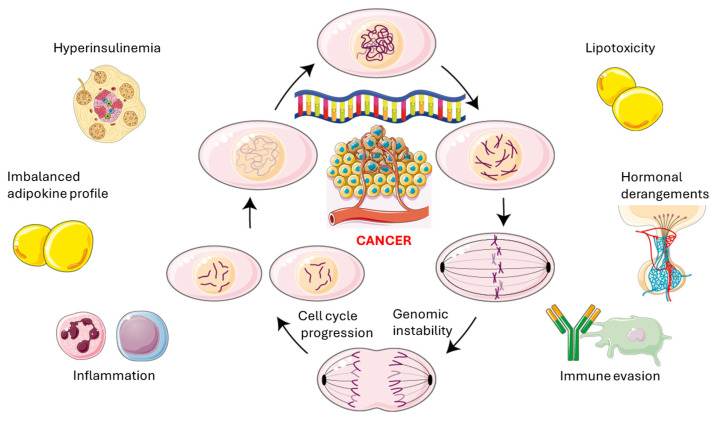
The self-reinforcing oncogenic cycle. Secondary hyperinsulinemia occurs within the context of and contributes to propagating genomic instability and cell cycle progression. This occurs alongside lipotoxicity, hormonal and adipokine imbalances, immune evasion and chronic inflammation. This original illustration was created using Servier Medical ART (SMART) and is based on references cited in the text. It is licensed under the Creative Commons Attribution 4.0 International License (CC BY 4.0).

Finally, IR-associated immune perturbations, characterized by predominance of M1 macrophages, Th17 skewing, and cytotoxic T-cell exhaustion, create an immunosuppressive environment that enables early tumors to avoid detection by the immune system [161]. This combination of factors, including hyperinsulinemia, imbalance of adipokines, lipotoxic-inflammatory stress, hormonal disruptions, and immune evasion, forms a self-reinforcing oncogenic cycle. This positions IR as a key molecular driver of cancer development, progression, and metastasis to various organs, as discussed in Section 9.2. Figure 4 illustrates the principal pathomechanisms involved in the association between IR and cancer.

### 9.2. Clinical Science Associating Insulin Resistance and Cancer

IR is linked to an increased risk of several types of cancer, including breast, liver, pancreatic, and colon cancer, through insulin acting as a growth factor. In association with insulin-resistance-related subclinical systemic inflammation, it can stimulate the growth and proliferation of cancer cells [162].

A seminal population-based case–control study conducted in 21,022 incident cases of 19 types of cancer and 5039 controls examined the association between obesity and the risks of various cancers [163]. Data have shown that obesity explained 7.7% of all cancers in Canada, 9.7% in men and 5.9% in women. Compared to non-obese controls those with a BMI ≥ 30 kg/m^2^ exhibited a higher risk of overall cancer [multivariable adjusted Odds Ratio (OR) = 1.34, 95% confidence interval (CI): 1.22, 1.48), non-Hodgkin’s lymphoma (OR = 1.46, 95% CI: 1.24, 1.72), leukemia (OR = 1.61, 95% CI: 1.32, 1.96), multiple myeloma (OR = 2.06, 95% CI: 1.46, 2.89), and cancers of the kidney (OR = 2.74, 95% CI: 2.30, 3.25), colon (OR = 1.93, 95% CI: 1.61, 2.31), rectum (OR = 1.65, 95% CI: 1.36, 2.00), pancreas (OR = 1.51, 95% CI: 1.19, 1.92), breast among postmenopausal women (OR = 1.66, 95% CI: 1.33, 2.06), ovary (OR = 1.95, 95% CI: 1.44, 2.64), and prostate (OR = 1.27, 95% CI: 1.09, 1.47).

Similarly, a recent nationwide study conducted in Hungary found that T2D was associated with a higher risk of cancer [164]]. The OR for overall cancer incidence among individuals with diabetes compared to non-diabetic controls was 2.50 (95% CI 2.46–2.55, *p* < 0.0001) with risks being significantly higher in males than in females [OR_males_: 2.76 (2.70–2.82) vs. OR_females_: 2.27 (2.22–2.33), *p* < 0.05 for male-to-female comparison].

Solid evidence indicates that MASLD plays an independent role in mediating these increased risks among individuals with conditions predisposing to IR [165,166,167,168,169].

Taken collectively, observational studies strongly support the theory that IR, as observed among individuals living with obesity and/or T2D, predisposes to the risk of cancer across various organ systems, with MASLD acting as a connecting link in this chain of events.

## 10. Principles of Treatment of Insulin Resistance

Given that (visceral) adipose tissue plays a key role in determining IR, it is expected that loss of body weight, regardless of how it is achieved, will invariably be followed by restored insulin sensitivity. Confirming this prediction, weight loss obtained through lifestyle changes or bariatric surgery is consistently associated with improved insulin sensitivity [170,171]. This improved insulin sensitivity can be so significant that it leads to T2D reversal or remission in a considerable number of individuals after metabolic surgeries such as sleeve gastrectomy and gastric bypass, which alter the anatomy and physiology of the upper digestive tract by restricting food intake and affecting calorie homeostasis [172,173]. However, bariatric and metabolic surgeries are invasive, carry risks of complications and long-term side effects [174].

Obesity-related IR can also be reduced with drugs. Currently, the threshold for utilizing insulin-sensitizing drugs includes prediabetes, obesity, diabetes, and PCOS. Metformin, the most commonly used drug treatment for decreasing IR in these settings, increases peripheral glucose utilization by inducing glucose transporter 4 expression and its increased translocation to the plasma membrane [175]. Additionally, Metformin has antioxidant and anti-inflammatory properties and improves lipid profiles [175]. Despite around 150 million people globally being treated with metformin, a drug that has been recommended as first-line therapy for T2D since 2009 due to its effective glucose-lowering activity, safety, and affordability, the exact mechanisms of action and critical targets of this drug remain incompletely defined [175]. Among the limitations of this drug, it is worth noting that metformin does not reduce cancer incidence in individuals with high BMI and/or altered glucose metabolism [176], nor does it improve liver histology in MASH [177].

Thiazolidinediones are considered the only true insulin-sensitizing antidiabetic drugs. One drug in this class, pioglitazone, has been shown to reduce cardiovascular events and slow the atherosclerotic process in high-risk patients with T2D [122]. By targeting nuclear receptors, this class of drugs improves the utilization of insulin in target organs, ultimately leading to an increased response to insulin and decreased circulating levels of glucose, triglycerides and free fatty acids [178]. However, some side effects associated with thiazolidinediones, such as fluid retention, weight gain, and an increased risk of fractures, make this class less suitable for elderly individuals. Other potential side effects include edema, worsening heart failure, and, in rare cases, liver toxicity and bladder cancer. More common, but less severe, side effects may include headaches, muscle pain, and upper respiratory tract infections [178].

Semaglutide, belonging to the class of glucagon-like peptide receptor 1 agonists (GLP-1RA) inhibits food intake by acting both centrally on neuronal circuits involved in hunger and reward and peripherally by slowing gastric emptying [179]. Semaglutide is available for weekly subcutaneous administration or daily oral administration [180]. It induces an average weight loss of 11.62 kg compared to placebo, reduces waist circumference, blood pressure, fasting blood glucose, C-reactive protein, improves lipid profiles, offers cardiovascular benefits for patients with established atherosclerotic CVD, reduces the odds of CKD and cardiovascular mortality, may resolve MASH and positively impact mental health and quality of life [152]. The most common adverse events are generally transient mild-to-moderate gastrointestinal complaints; hypoglycemia is more common without lifestyle intervention and weight regain will often follow semaglutide withdrawal [179].

Resmetirom, approved as a thyroid hormone receptor-β (THR-β) agonist for fibrosing MASLD, enhances lipophagy, mitophagy, mitochondrial biogenesis, and fatty acid β-oxidation [181]. It increases deiodinase type 1 expression, boosting local T4-to-T3 conversion while lowering inactive reverse T3 and CPT1, which supports oxidative phosphorylation, reduces ROS, and improves insulin sensitivity [180]. The drug also demonstrates anti-inflammatory and anti-fibrotic effects by inhibiting nuclear factor κB (NF-κB), Janus kinase- signal transducer and activator of transcription 3 (Jak-STAT3), tumor necrosis factor-α (TNF-α), interleukin -6 (IL-6), Kupffer cell activation, and transforming growth factor-β (TGF-β) signaling [182]. Additionally, resmetirom improves lipid profiles and raises SHBG, suggesting both hepatic and broader metabolic benefits, with potential impacts on insulin sensitivity and diabetes risk that deserve further investigation [183].

SGLT2 inhibitors have been shown to reduce cardiovascular events in high-risk patients with T2D, but their cardiovascular benefit appears to be mediated via mechanisms other than reduced IR [121]. Additional drug agents of potential significance in combating IR have been discussed elsewhere [140,184].

## 11. Conclusions and Research Agenda

Originally described in the diabetes arena, IR has now become a key player in common non-communicable diseases such as obesity, metabolic dysfunction, cardiovascular-hepato-nephro-metabolic health, and cancer. Reflecting the complexity of lifestyle habits, such as dietary preferences and variable attitudes towards engaging in physical activity, which are intimately associated with psychosocial causes, IR mirrors the challenges faced by developed and developing countries in terms of food security and social equity [185,186] as key determinants of health and disease. However, IR is also strongly dependent on physiological processes and is affected by sex and aging. From a therapeutic point of view, the lesson from MASH is that combating IR may be a “necessary but not sufficient” condition [187] as this physiopathological derangement may spark a dysmetabolic fire that will autonomously self-maintain over time.

Consequently, the next decade demands a concerted, transdisciplinary research agenda. This agenda should focus on (i) deciphering the spatiotemporal dynamics of IR across organs by integrating single-cell multi-omics and advanced in vivo imaging, (ii) mapping the bidirectional crosstalk among hepatokines, myokines, adipokines, and the immune system using CRISPR-based perturbation screens, (iii) developing “digital twins” capable of simulating individual metabolic trajectories and predicting therapeutic responses, (iv) testing combination metabolic therapies such as GLP-1RA + SGLT2 inhibitors and THR-β agonists in adaptive platform trials with clamp-based and imaging-based IR read-outs, (v) exploring microbiome-targeted interventions such as synbiotics to attenuate gut-derived metaflammation, (vi) designing precision-lifestyle interventions driven by wearables, continuous glucose monitoring, and AI-guided feedback loops, and (vii) embedding socio-ecological studies that evaluate how food-insecurity mitigation, urban-design innovations, and fiscal policies modulate population-level IR.

An improved understanding of the molecular events underlying IR and the pathobiology of β-cell exhaustion promises to provide innovative approaches to clinically heterogeneous disease phenotypes spanning common non-transmissible chronic conditions from diabesity, cardiovascular disorders, organ dysfunction and cancer.

## Figures and Tables

**Table 1:** Etiology of acquired insulin resistance [22,23,24,25,26,27,28,29,30,31,32,33,34,35,36,37,38,39,40,41,42,43,44,45,46,47,48,49,50,51,52,53,54,55].

	Model	Mechanisms	Metabolic Effects	Ref.	Comment
**Physiological**					
***Prolonged fasting***	Lean and obese men.	Markedly increased levels of β-hydroxybutyrate and β- hydroxybutyrylcarnitine in skeletal muscle.	Substrate competition due to increased metabolism of ketone bodies rather than oxidation of long-chain fatty acids.	[22]	Prolonged fasting should be avoided to prevent IR.
***Strenuous exercise***	Healthy volunteers.	Mitochondrial functional impairment	Impaired glucose tolerance	[23]	Excessive exercise training should be avoided to prevent IR.
***Puberty***	Healthy, normal-weight, prepubertal children.	Adiponectin levels were halved, partially mediated by increased GH secretion.	Increased total body lipolysis and decreased glucose oxidation.	[24]	Balanced diet and non-strenuous physical activity may be a valuable approach for preventing puberty-related IR.
***Pregnancy***	Pregnant and GDM women during the third trimester.	hPGH increases the expression of the p85α subunit of PI3K in skeletal muscle, which, in turn, acts as a dominant-negative competitor to forming a PI3K heterodimer with the p110 subunit, thus inhibiting the PI3K activity and attenuating the downstream insulin signaling.	Increased risk of GDM	[25]	Balanced diet and moderate physical activity should be suggested for preventing IR in pregnancy.
***Aging***	Individuals with normal FPG and BMI values.	Age is negatively related to IR and GE in both sexes.	The risk of T2D increases with age.	[26]	Normal weight should be maintained during ageing with appropriate lifestyle habits to prevent age-related IR.
**Pathological**					
***Obesity***	Humans	Ectopic accumulation of lipotoxic intermediates such as DAGs, Ceramides, and LPA.	Obesity is a major risk factor for incident T2D.	[27]	Obesity should be treated with appropriate lifestyle habits, drug treatment and bariatric surgery whenever indicated.
***Physical inactivity***	Humans	Impaired muscle glucose uptake, altered lipid metabolism, oxidative stress, inflammation, and endothelial dysfunction	Sedentary behavior is a major risk factor for incident T2D.	[28]	Sedentary behavior should be discouraged at the general population level through appropriate public health policies.
***Altered sleep–wake cycle***	Healthy non-obese subjects	Reduced β-cell secretory response and increased NEFAs.	Sleep deprivation is a risk factor for an increase incidence of T2D.	[29]	Healthy sleep habits should be promoted as part of public health strategies for preventing T2D.
***Smoking***	Muscle cell culture.	Nicotine induces IR in skeletal muscle by activating mTOR.	Tobacco smoking increases the risk of T2D.	[30]	Public health campaigns and specific tax policies should be implemented to discourage smoking.
***Stressful conditions***					
*Trauma*	SIH individuals compared to NDN, DN, and DH patients.	SIH patients had elevated IL-6 concentrations relative to NDN, DN, and DH patients.	SIH is linked to higher mortality in trauma.	[31]	Prompt treatment of SIH is a rational approach to reduce mortality in the context of major trauma.
*Surgery*		Postoperative IR results from muscle inflammation and reduced suppression of FOXO1-driven PDH kinase-4 mRNA and protein expression after surgery.	Reduced oxidation of glucose, resulting in impaired glucose uptake in muscle.	[32]	Preoperative carbohydrate supplementation may limit muscle inflammation while improving the inhibition of PDH kinase-4 activity mediated by insulin.
*DKA*	Adult patients with diabetes.	Absolute insulin deficiency, increased counter-regulatory hormones, and a surge in pro-inflammatory cytokines.	Increased lipolysis determines the release of FFAs from the adipose tissue into the bloodstream and unrestrained hepatic fatty acid oxidation in the liver leads to ketone bodies	[33]	Key steps to prevent DKA include consistently monitoring glycemic levels, adhering to prescribed antidiabetic medications, assessing for ketones (if blood glucose exceeds 250 mg/dL or during illness) and maintenance of adequate hydration status.
*High-sodium diets*	Humans	Overproduction of fructose and ghrelin, leptin resistance and IR, reduced circulating levels of adiponectin and GLP-1. These may eventually facilitate obesity development via increased food intake and expanded WAT.	High-salt intake is closely linked to CVD, especially HTN and, as suggested by emerging evidence, also to metabolic disorders.	[34]	Limiting salt intake is an important recommendation to promote cardiovascular health.
***Organ failure***					
*Uremia*	Humans with CKD of variable severity.	Uremic toxins disrupt insulin signaling, reducing glucose uptake even with normal insulin, due to inflammation, oxidative stress, and inhibitory molecules that degrade proteins like IRS-1, ultimately impairing glucose metabolism.	IR, commonly observed among individuals with ESRD, also occurs in CKD patients with minimally increased creatinine serum levels.	[35]	Treating IR in CKD is critical to slowing the progression to ESRD, reducing the odds of CVD, and decreasing the risks of fluid/electrolyte imbalances and infections. To achieve this goal, it is recommended to manage underlying mineral bone disorders, enhance dialysis adequacy, implement appropriate lifestyle modifications, and consider the use of medications such as ACE inhibitors.
*Liver cirrhosis*	Humans	Hyperinsulinemia results from impaired muscular glucose uptake and liver dysfunction eventually causing defective glucose storage and defective signaling in peripheral tissues.	The primary contributing factors are inflammation, lipotoxins, sarcopenia, intestinal dysbiosis, and chronic hyperinsulinemia.	[36]	Correcting IR in cirrhosis is crucial to prevent complications such as HCC, infections, CKD, and decrease mortality. The therapeutic strategy involves diet and exercise, alongside medications such as (cautiously) Metformin, TZDs, DPP-4i, and GLP-1RA, with liver transplantation often restoring NGT.
**Infections**					
*Sepsis*	Humans	Inhibition of tyrosine kinase activity in the beta subunit, enhanced proteolytic activity leading to receptor loss from the plasma membrane, and the potential translocation of insulin receptors into the nucleus, where they may associate with gene promoters.	In sepsis, hyperglycemia is caused by impaired insulin receptor function, decreased GLUT4 translocation, activation of inflammatory JNK1 signaling, increased *lipolysis*, reduced tissue glucose uptake, and elevated hepatic glucose production.	[37]	Correcting hyperglycemia in sepsis is crucial to prevent impaired immune responses, reduced WBC functionality, organ dysfunction, and the increased risk of mortality due to metabolic stress and systemic inflammation.
*HIV*	Humans	Macrophages present within adipose tissue secrete inflammatory cytokines such as TNF-alpha and IL-6 that are likely mediators of IR in HIV infection.	HIV individuals are exposed to the risk of T2D	[38]	The management of IR in HIV-infected individuals involves dietary modifications, regular physical activity, and pharmacological interventions. Available drugs include Metformin (which requires careful monitoring with some ART agents), medications to reduce visceral fat, and possibly TZDs, DPP-4i, or angiotensin receptor blockers. Specific ARTs that may cause IR should also be addressed.
**Endocrinological**					
*PCOS*	Humans	IR in PCOS arises due to a shift from normal tyrosine phosphorylation to impaired serine phosphorylation of the insulin receptor and IRS proteins, thereby disrupting the PI3K/Akt pathway essential for glucose uptake.	IR in PCOS disrupts glucose and lipid metabolism, resulting in hyperinsulinemia. This further stimulates increased androgen production contributing to menstrual irregularities and infertility, while also significantly elevating the risk for T2D, MetS, MASLD, and CVD.	[39]	Dietary modifications, physical activity, weight reduction, and inositol supplementation—with or without insulin-sensitizing medications—are implemented to improve insulin sensitivity, decrease hyperandrogenism, restore ovulatory function, and address metabolic dysfunction.
*Cushing’s syndrome*	Humans	IR owing to hyper-cortisolemia results from blocked GLUT4 translocation, inhibited glycogen synthase, increased hepatic gluconeogenesis, and stimulated lipolysis that further impairs insulin action through high FFAs levels.	Cushing’s syndrome causes a wide spectrum of metabolic derangements due to excess cortisol, primarily manifesting as MetS.	[40]	Managing IR in Cushing’s syndrome involves addressing the root cause, hyper-cortisolemia, with medications like metyrapone or mifepristone, alongside lifestyle changes such as diet, exercise, and weight loss. Specific diabetes drugs should be used to lower glycemia, with lifestyle adjustments being crucial for long-term prevention and management.
*Acromegaly*	Humans	An excess of GH antagonizes insulin action at the level of liver, skeletal muscle, and adipose tissue via multiple pathways, most prominently by enhancing lipolysis and disrupting insulin signaling at the post-receptor level.	Acromegaly leads to a variety of cardiometabolic disorders, including HTN, IR, T2D atherogenic dyslipidemia OSA and accelerated CVD. Driven by GH and IGF-1, these conditions raise the risk of heart failure, arrhythmias and early cardiovascular death.	[41]	Treating IR in acromegaly involves targeting the underlying GH excess through surgery or medications to normalize GH/IGF-1 levels, which significantly improves glucose metabolism. Additionally, standard diabetes management is crucial, with pegvisomant offering unique benefits in improving IR irrespective of weight loss [42].
***Drug-induced***					
*Long-term glucocorticoids*	Humans, animal models and cell culture studies	Enhanced gluconeogenesis and endogenous glucose production.	Glucocorticoids induce hyperglycemia, glucose intolerance and steroid-induced diabetes, weight gain with deleterious fat redistribution, and increased levels of circulating FFAs, sarcopenia, and osteoporosis	[43]	The prevention and management of glucocorticoid-induced IR primarily focus on dietary modifications, regular physical activity, and insulin-sensitizing drugs.
*Cyclosporine A, Syrolimus*	In vivo rat model.	Reduced expression of genes (IRS-1, Glut4, and Glut1), which are associated with insulin action and glucose uptake and upregulation of genes and/or proteins involved in hepatic lipogenesis and gluconeogenesis, accompanied by a decrease in these factors in adipose tissue.	Cyclosporin A and sirolimus are immunosuppressive agents that have been linked to the development of dyslipidemia, IR, and new-onset diabetes following transplantation.	[44]	Maintaining a healthy lifestyle, avoiding high-risk medications, and controlling glucose early help preserve insulin sensitivity in individuals undergoing immunosuppressive pharmacotherapy.
*Niacin* (nicotinic acid; vitamin B3)	Rats	Niacin primarily determines IR through activation of its receptor GPR109A, which reduces insulin signaling in adipose tissue by downregulating IRS-1, PDE3B, and β-adrenergic receptors, thereby decreasing lipolysis. In pancreatic beta-cells, GPR109A activation increases ROS, raising UCP2 and PPARγ, and impairs glucose-stimulated insulin secretion.	Niacin, while vital for energy metabolism, DNA repair and improving atherogenic dysplidemia, also increases glycemic levels, causes flushing, and promotes inflammation through its breakdown products, making its therapeutic use controversial when taken in excess.	[45]	The combination of niacin with regular exercise may potentially improve glycemic control, lower FFAs and lessen negative effects on insulin sensitivity.
*Beta-adrenergic blocking agents*	Mammalian cells	Prolonged activation of the cAMP/PKA pathway can disrupt insulin signaling due to negative crosstalk mechanisms, including PKA-mediated serine phosphorylation of both the IRS and Akt. This process inhibits PI3K/Akt-dependent glucose uptake by preventing GLUT4 translocation.	Beta-blockers induce a diverse metabolic spectrum, primarily by slowing metabolism, impairing glucose/lipid mobilization, potentially causing weight gain/body fat increase, and blunting hypoglycemia symptoms, though carvedilol may offer benefits like reduced IR and antioxidant effects.	[46]	Use vasodilating beta-blockers like carvedilol, monitor glucose and lipids, adjust diet or medications as needed, educate about hypoglycemia, and consider SGLT-2i or GLP-1RAs for high-risk patients. Avoid stopping treatment abruptly.
*Protease inhibitors*	Humans	Acute inhibition of GLUT4-mediated glucose transport, and defective insulin signaling account for IR whereas interference with adipogenesis and adipocyte apoptosis and activation of lipolysis are potential mechanisms of drug-induced lipodystrophy among individuals exposed to Protease inhibitors.	HIV Protease inhibitors are associated with the pathogenesis of IR, dyslipidaemia, lipodystrophy and atherosclerosis, described with highly active ART therapy.	[47]	Preventing and managing HIV protease inhibitors involves maintaining healthy diet, regular physical activity, weight reduction when necessary, carefully selecting ART therapy, and consistently monitoring glycemic values.
*SRRI*	Mouse model.	SSRIs can cause IR by disrupting insulin signaling, mainly through kinase activation that phosphorylates IRS proteins. This impairs insulin function, reduces glucose-stimulated insulin secretion from pancreatic beta cells, and may trigger ER stress and beta-cell apoptosis, contributing to T2D.	SSRI use may cause metabolic changes, mainly due to weight gain from increased appetite, which can contribute to MetS, though clinical impact varies among patients.	[48]	To prevent and manage SSRI-related metabolic dysfunction, monitoring body weight, lipids, and glucose s is important. It is also recommended to follow a healthy diet and exercise regimen, consider SSRIs with lower metabolic risks, and adjust treatment as needed under medical supervision.
*Atypical antipsychotics*	Humans	Atypical antipsychotics can cause IR by directly interfering with insulin signaling in myocytes, adipocytes, and hepatocytes.	Atypical antipsychotics promote weight gain that harms pancreatic beta-cells and disrupt neurotransmitter systems involved in glucose and appetite regulation, which may lead to hyperglycemia and MetS.	[49]	The longer the duration of treatment, the higher the risk of developing IR [50].
*Androgen-deprivation therapy*	Mouse models	Global deletion of androgen receptor in male mice results in the characteristics of MetS featuring IR owing to reduced ability of insulin to stimulate activation of downstream PI3K in skeletal muscle and liver. Leptin resistance was also demonstrated with deregulated food intake and increased body weight.	Anti-androgenic therapy leads to sarcopenic obesity, which increases the risk of IR, hyperglycemia, T2D, atherogenic dyslipidemia, CVD, and osteoporosis-related fractures.	[51,52]	To prevent and treat IR from anti-androgen therapy, diet, exercise, and weight loss should be prioritized while considering insulin-sensitizing medications in combination with anti-androgens for high-risk individuals to mitigate metabolic risks linked to hypotestosteronemia.
*Statins*	Mouse model	Statin-induced IR results from disruption of the mevalonate pathway, impairing insulin signaling in liver, muscle, and pancreatic cells; it can also affect lipid metabolism, mitochondrial function, gluconeogenesis, and inflammation.	Statin therapy can cause metabolic dysfunction, including IR, new-onset diabetes, muscle complaints, often linked to lipophilic statins and high doses, affecting CoQ10 levels and gut microbiota, though benefits usually outweigh risks for cardiovascular health	[53]	CoQ10 supplementation in individuals taking statins is associated with a reduced risk of NOD, independent of the CoQ10 dose [54].
*Insulin therapy*	Humans	Exogenous insulin antibody syndrome is a rare cause of extreme IR.	Exogenous insulin therapy, while vital, introduces its own metabolic risks, comprising hypoglycemia, weight gain, fluid retention, and exacerbating underlying IR. A spectrum of dysmetabolic features will potentially ensue (e.g., dyslipidemia, inflammation, HTN, localized lipodystrophy, and increased CVD risk).	[55]	Treatment with high-dose methylprednisolone and mycophenolate mofetil, followed by a tapering regimen of prednisone, resulted in a significant improvement in a reported case of extreme IR [55].

**List of abbreviations and definitions used**: ACE—angiotensin-converting enzyme; ART—anti-retroviral; BMI—body mass index; counter-regulatory hormones-glucagon, cortisol, catecholamines; cAMP/PKA—cyclic AMP/Protein Kinase A; CKD—chronic kidney disease; CoQ10—coenzyme Q10; CVD—cardiovascular disease; DAGs—diacylglycerols; DKA—Diabetic ketoacidosis; DH—diabetic hyperglycemia; DN—diabetic normoglycemia; DPP-4i—dipeptidyl peptidase-4 inhibitors; ER—endoplasmic reticulum; ESRD—end-stage renal disease; FFA—free fatty acids; FPG—fasting plasma glucose; GDM—gestational diabetes mellitus; GE—glucose effectiveness; GH—growth hormone; HDL—high density lipoproteins; HIV—human immunodeficiency virus; hPGH—human placental growth hormone; GLP-1 -glucagon-like peptide 1; GLUT4—glucose transporter 4; GPR—G protein-coupled receptor; HCC—hepatocellular carcinoma; HTN—arterial hypertension; IGF-1—insulin-like growth factor 1; IL—interleukin; IR—insulin resistance; IRS- 1 insulin receptor substrate-1; ketone bodies-β-hydroxybutyrate and acetoacetate; JNK1—janus kinase 1; LPA—lysophosphatidic acid; MASLD—metabolic dysfunction-associated steatotic liver disease; MetS—metabolic syndrome; mRNA—messenger RNA; mTOR—mammalian target of rapamycin; NDN—non-diabetic normoglycemia; NEFAs—non-esterified fatty acids; NGT—norma glucose tolerance; OSA—obstructive sleep apnea, PCOS—polycystic ovary syndrome; PDH—pyruvate dehydrogenase; PI3K—phosphatidylinositide-3 kinase; RNA—ribonucleic acid; SGLT-2 inhibitors; SRRI serotonin receptor reuptake inhibitors; TNF—tumor necrosis factor; T2D—type 2 diabetes; TZDs—Thiazolidinediones; WAT—white adipose tissue; WBC—white blood cells.

## Data Availability

No new data were created or analyzed in this study. Data sharing is not applicable to this article.

## Abbreviations

The following abbreviations are used in this manuscript:

BMI	body mass index
CKD	chronic kidney disease
CI	confidence interval
CPT1	carnitine O-palmitoiltransferase 1
CVD	cardiovascular disease
DAMP(s)	damage-associated molecular pattern(s)
GLP-1RA	glucagon-like peptide receptor 1 agonist(s)
HOMA-IR	homeostasis model of insulin resistance
IL-6	interleukin-6
IR	insulin resistance
IRR	incidence rate ratio
ISR1/2	insulin receptor substrate 1/2)
IST	insulin tolerance test
MACE	major adverse cardiovascular events
MASLD	metabolic dysfunction-associated steatotic liver disease
MASH	metabolic dysfunction-associated steatohepatitis
mtDNA	mitochondrial DNA
OR	odds ratio
PCOS	polycystic ovary syndrome
QUICKI	quantitative insulin sensitivity check index
ROS	reactive oxygen species
SGLT2	sodium-glucose transporter 2
SHBG	sex hormone binding globulin
SRRI	selective serotonin reuptake inhibitors
THR-β	thyroid hormone receptor-β
T2D	type 2 diabetes
TLR4	toll-like receptor 4
TyG	triglyceride-glucose index
